# Perceptions of mistreatment among trainees vary at different stages of clinical training

**DOI:** 10.1186/s12909-016-0853-4

**Published:** 2017-01-14

**Authors:** Afif N. Kulaylat, Danni Qin, Susie X. Sun, Christopher S. Hollenbeak, Jane R. Schubart, Antone J. Aboud, Donald J. Flemming, Peter W. Dillon, Edward R. Bollard, David C. Han

**Affiliations:** 1 Department of Surgery, The Pennsylvania State University, College of Medicine, P.O. Box 850, 500 University Drive, H137, Hershey, PA 17033-0850 USA; 2 School of Labor and Employment Relations, The Pennsylvania State University, University Park, PA USA; 3 Department of Public Health Sciences, The Pennsylvania State University, College of Medicine, Hershey, PA USA; 4 Department of Radiology, The Pennsylvania State University, College of Medicine, Hershey, PA USA; 5 Department of Medicine, The Pennsylvania State University, College of Medicine, Hershey, PA USA

**Keywords:** Clinical learning environment, ACGME, Hidden curriculum, Professionalism, Core competencies, Milestones, Burnout

## Abstract

**Background:**

Mistreatment of trainees remains a frequently reported phenomenon in medical education. One barrier to creating an educational culture of respect and professionalism may be a lack of alignment in the perceptions of mistreatment among different learners. Through the use of clinical vignettes, our aim was to assess the perceptions of trainees toward themes of potential mistreatment at different stages of training.

**Methods:**

Based on observations from external experts embedded in the clinical learning environment, six thematic areas of potential mistreatment were identified: verbal abuse, specialty-choice discrimination, non-educational tasks, withholding/denying learning opportunities, neglect and gender/racial insensitivity. Corresponding clinical vignettes were created and distributed to 1) medical students, 2) incoming interns, 3) residents/fellows. Perceptions of the appropriateness of the interactions depicted in the vignettes were measured on a 5-point Likert scale. Scores were categorized into neutral or appropriate (≤3) or inappropriate (i.e. mistreatment) (>3) and compared using chi-squared tests.

**Results:**

Four hundred twenty seven trainees participated (182 students, 120 interns, 125 residents/fellows). Proportions of students perceiving mistreatment differed significantly from those of interns and residents/fellows in domains of verbal abuse, specialty discrimination and gender/racial insensitivity (*p* < 0.05). In scenarios comparing interns to residents/fellows, no significant differences were noted in perceptions of mistreatment in the domains of non-educational tasks, withholding learning and neglect.

**Conclusions:**

Perceptions of mistreatment differ at different developmental stages of medical training. After exposure to the clinical learning environment, perceptions of incoming interns did not differ from those of residents/fellows, implicating clinical rotations as a key period in indoctrinating students into the prevailing culture. More longitudinal studies are needed to confirm or better examine this phenomenon.

**Electronic supplementary material:**

The online version of this article (doi:10.1186/s12909-016-0853-4) contains supplementary material, which is available to authorized users.

## Background

Professionalism is a core competency of the Accreditation Council on Graduate Medical Education (ACGME), and a critical component of the Clinical Learning Environment Review (CLER) [1]. Professionalism is central to creating a culture of respect, providing the foundation for an optimal educational environment for trainees [2–4]. However, differences in perception among learners on what constitutes mistreatment may present a barrier to fostering this culture.

In promoting a professional learning environment, addressing mistreatment remains an enduring challenge in medical training [5–16]. Much of the study of mistreatment in medical training has been based on the Association of American Medical Colleges (AAMC) Graduation Questionnaire [5]. The prevalence of reported mistreatment among graduating medical students has consistently ranged between 17 and 20% annually [5]. Beyond hampering the clinical learning environment (CLE), the negative consequences of this behavior on trainees have been well documented [6–13]. Some authors have conceded that efforts to eradicate learner mistreatment have been hindered by the effect of the “hidden curriculum [14]”. Others have highlighted the “eye of the beholder”, describing the spectrum of differing perspectives engendered by the term “mistreatment [12]”. Greater understanding of this issue has become a priority of both the AAMC and the American Medical Association [5]. Accordingly, the AAMC proposed five major domains requiring further study, evidence and discussion: 1) a definition of mistreatment, 2) the reporting of mistreatment, 3) the assessment of alleged mistreatment, 4) any intervention after mistreatment was identified, and 5) the prevention of mistreatment [5].

Within this framework, we sought to characterize our organizational culture around the issue of learner mistreatment [17, 18]. The present study has implications for both the “definition of mistreatment”, and the “assessment of alleged mistreatment”. In this pilot experience, our aims were to 1) use clinical vignettes to depict institution-specific interactions that might potentially represent mistreatment and 2) assess perceptions of mistreatment among medical trainees. We hypothesized that learners’ perceptions of mistreatment change as they advance in clinical training.

## Methods

This study was reviewed by the Penn State College of Medicine (PSCOM) Institutional Review Board and determined to be exempt from formal review.

The Culture of Respect in Education (CORE) initiative was developed as part of an institutional effort to improve the CLE at PSCOM [19]. Addressing learner mistreatment was identified as an initial target to promote CORE, with the aim of clarifying and aligning perceptions of mistreatment between trainee expectations and organizational culture. To obtain external expertise in workplace environment professionalism, this process was developed and implemented with the Penn State School of Labor and Employment Relations (SLER). A graduate student, mentored by experts in SLER, was embedded within our patient care teams for a period of 8 weeks, and observed the interactions between members of the healthcare team on the wards, clinics, conferences and operating rooms. As an observer without prior healthcare experience, this individual provided us with an external lens into our CLE.

**Fig. 1:**
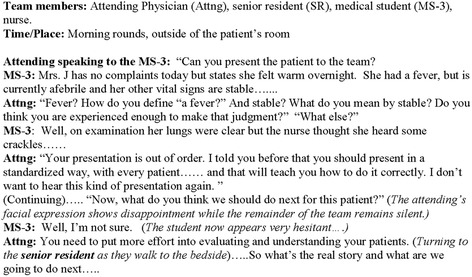
Example of vignette (Verbal abuse/public humiliation)

Based on this experience, six institution-specific thematic areas of potential learner mistreatment were identified: 1) Verbal abuse/public humiliation, 2) Specialty discrimination, 3) Performing non-educational tasks, 4) Withholding/denying learning opportunities, 5) Neglect, 6) Gender/racial insensitivity. Corresponding paper-based clinical vignettes were developed between SLER faculty and senior clinicians including the Designated Institutional Official and Vice-Chair for Education in the Department of Surgery (see Fig. 1 and Additional file 1).

These vignettes were delivered to trainees as part of their onboarding process or curricular training. For students, training was delivered prior to commencement of clinical rotations. For interns, training was delivered prior to assuming clinical responsibilities. For residents/fellows, this exercise was incorporated into their Graduate Medical Education (GME) curriculum. Trainees were asked to rate the interactions depicted in the scenarios on a 5-point Likert scale where “1” represented completely appropriate behavior, “3” represented neutral behavior and “5” represented completely inappropriate behavior. The response rate was 100% among attendees. Medical student and intern attendance comprised 100% of the total possible attendance, while resident/fellow attendance comprised 26% (125/478) of the total possible attendance.

Likert responses were dichotomized into inappropriate (i.e. mistreatment) (score of greater than three) or neutral/appropriate (scores of three or less). Proportions of learners reporting mistreatment in the evaluated clinical vignettes were compared across groups of learners with chi-squared tests. The vignettes addressing the themes of “verbal abuse/public humiliation”, “gender/racial insensitivity” and “specialty discrimination” were delivered to all learners. The vignettes addressing the themes of “neglect”, “withholding/denying learning opportunities” and “performing non-educational tasks”, were modified for delivery to the medical students, and therefore, were not included in the analysis for these themes. All analyses were performed with STATA software (version 10/MP; Stata Corp., College Station, TX). *p* values < 0.05 were considered significant.

## Results

**Fig. 2:**
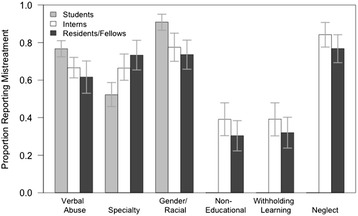
Proportions of learners reporting perception of mistreatment across case vignettes. Error bars reflect 95% confidence intervals

There were 427 trainees that participated (182 students, 120 interns, 125 residents/fellows). Proportions of learners reporting mistreatment across clinical vignettes is presented graphically in Fig. 2. Across the three clinical vignettes (verbal abuse/public humiliation; specialty discrimination; gender/racial insensitivity) in which students, interns and resident/fellows were compared, there was a significant difference in the perceptions of students when compared to both interns and to residents/fellows (*p* < 0.05 for all). In contrast, the perceptions between interns and residents/fellows were not significantly different for these three scenarios. In the remaining three scenarios (neglect; withholding of learning opportunities; and performing non-educational tasks) where interns and resident/fellows were compared, there were no significant differences noted in their overall perceptions of mistreatment in the aforementioned clinical vignettes. Additionally, in the scenario describing “specialty discrimination”, in contrast to the other scenarios, there was an increased perception of mistreatment among interns and residents/fellows when compared to students. Finally, in the scenarios describing “performing non-educational tasks” and “withholding/denying of learning opportunities” less than 50% of learners identified these scenarios as depicting mistreatment, relating that the majority found these scenarios to be neutral or appropriate interactions.

## Discussion

When evaluating our trainees’ perceptions of mistreatment using clinical vignettes, we found that the perceptions of mistreatment among students differed significantly from interns and residents/fellows across various clinical vignettes. Furthermore, when comparing perceptions of mistreatment among incoming interns and residents/fellows, there were no significant differences. Additionally, there were themes such as “performing non-educational tasks” and “withholding/denying learning opportunities” where the majority of respondents found these scenarios to represent neutral or appropriate behavior rather than mistreatment or inappropriate conduct. Summatively, these findings highlight discordant perceptions of mistreatment among trainees at different levels in certain themes, with more concordant perceptions of mistreatment in others. Furthermore, the similarity between the perceptions toward mistreatment among incoming interns and residents/fellows when compared to medical students implicates the CLE as a key period in influencing or shaping these perceptions.

In a study using video-based clinical vignettes to identify what constitutes medical student abuse among medical students, residents, nurses and attending physicians, Ogden et al found that these groups generally agreed upon what constitutes abuse [20]. The themes selected for their study, “belittlement”, “ethnic insensitivity”, “negative feedback”, “sexual harassment” and “excluding from a learning opportunity” closely parallel themes identified at our institution and others, demonstrating the pervasiveness of these issues [5, 14, 21, 22]. Similar to our study, in the scenarios of “belittlement”, “ethnic insensitivity” and “sexual harassment”, these themes were generally perceived to represent mistreatment. Likewise, in the scenario where a medical student was excluded from witnessing a procedure, respondents did not categorize this as an example of mistreatment.

Other studies have highlighted a “hidden curriculum” or environmental factors that may undermine efforts to address mistreatment. Over a 13-year period, researchers at UCLA adopted a proactive and multifaceted approach to address these components and eradicate medical student mistreatment [14]. However, the incidence of mistreatment, thematic patterns and severity of episodes, remained constant throughout the endeavor. The results of our study suggest that exposure to the clinical environment indoctrinates students to the prevailing organizational culture. This was evident in our study as students prior to their clinical rotations harbored certain perceptions of mistreatment; however, after exposure to the clinical environment, and on matriculation as an intern (prior to any clinical intern rotations), incoming interns already shared attitudes that were more similar to those of residents/fellows than those of students.

A great deal of literature has emerged on the pervasiveness of burnout in medical training [8, 23–26]. Burnout has been associated with emotional exhaustion, lower empathy, depersonalization of care and decreased professionalism [8, 23–26]. Higher proportions of burnout have been demonstrated in medical students, residents and early career physicians compared to the general population, and in a study of US surgical residents, nearly 70% met the criteria for burnout in one of the studied inventories [25, 26]. One study demonstrated a strong association between burnout and the prevalence of mistreatment, particularly recurrent episodes of mistreatment [8]. Factors within the learning and work environment, rather than individual attributes, are the major drivers of burnout [24]. In this context, our findings may represent a desensitization or depersonalization toward issues of mistreatment among incoming interns and residents/fellows who have already been indoctrinated into the clinical learning environment compared to students.

Perception of mistreatment is a complex issue, providing measurable challenges in addressing mistreatment among trainees [5, 12, 14, 16, 17]. Medical students must transition from being the center of the educational process during their initial years of training, to a patient- and relationship-centered training paradigm where patients, families and their care take priority. Fried et al postulated that as students progress through their training, they may be better adjusted and understand their role in a complex health care system [14]. This evolution or maturation of attitudes may explain the findings of the present study, which also extends beyond medical students to post-graduate trainees.

When qualitatively assessing medical students’ perceptions of mistreatment, Gan et al proposed that learners may be referring to a spectrum with blatant episodic occurrences of mistreatment (e.g. physical abuse, sexual discrimination) on one end and a suboptimal learning environment on the other end [12]. While the former was rare and generally well-encompassed by institutional mistreatment policies, the latter was felt to be more distressing by students and included: feeling undervalued, having limited voice, performing work without perceived educational value, and a lack of a sense of belonging within the structure of the medical team [12].

Collectively, these studies expand our understanding of mistreatment, and our data may provide further insight into the role of the CLE in evolving professional behaviors. While our data suggest that perceptions of mistreatment are different at various changes of clinical training, not all domains showed more tolerance of unprofessional behavior by learners at more advanced levels of training. Additionally, attitudes toward two vignettes were neutral, without any significant change across level of training. This may suggest either vignettes that did not adequately assess the domain, or that these domains have been adequately addressed at this institution. It may also be that these domains encompass themes that are more contraversial among trainees, or behaviors for which learners have a higher threshold to label as mistreatment. In aggregate, these data highlight the potentially institution-specific nature of a professionalism curriculum, focused on existing and desired institution-specific organizational culture.

## Limitations

There are limitations to consider in this pilot experience. The vignettes represent a collaborative effort between senior faculty in SLER and Graduate Medical Education, but are not validated instruments. While the themes identified in this study were consistent with prior research, through literature review, bias may have been introduced into the process of observation and cataloguing forms of mistreatment, potentially omitting and failing to identify “new” forms of mistreatment. Furthermore, the themes depicted in the clinical vignettes were obtained from field notes by the observer embedded in the CLE and in consultation with the aforementioned experts, but were not obtained through rigorous validated qualitative methods.

The sample size was limited, particularly amongst residents and fellows. While for students and interns the exercise was delivered as part of the onboarding process, for clinical residents and fellows, it was delivered as part of the graduate medical student curriculum which is not mandatory due to ongoing clinical responsibilities. This may introduce the potential for sampling bias. Half of the vignettes were modified to make them more accessible to the students, who were early in their clerkship experience. Consequently, we were unable to include the perceptions of these learners in the respective models for these themes. Furthermore, there were shifts in the level of training of the victim in the various scenarios. While this was intended to provide diversity to the scenarios, it may also have inadvertently introduced an additional bias related to group membership, sympathy and interpersonal relatedness of respondents evaluating the clinical vignettes. Finally, the themes of mistreatment and trainee perceptions identified relate specifically to an experience at a single academic institution and may not reflect the experiences of other institutions.

## Conclusions

Perceptions of mistreatment differ between levels of clinical trainees, with perceptions that appear to change after exposure to the clinical learning environment. Clinical rotations during medical school play a key role in indoctrinating students into the prevailing medical culture. It is unclear how and to what extent the CLE and the often cited “hidden curriculum” are responsible for these differing perspectives at different stages of clinical training. Focusing on this transition period in student training, as well as uncovering the underlying factors, such as burnout, that may result in desensitization or depersonalization of more experienced clinical trainees or faculty to matters of mistreatment will be important steps in changing medical culture and improving the CLE. As such, refinement of these clinical vignettes, further examination of the CLE, as well as larger and longitudinal studies, would be of significant benefit in better understanding the issue of learner mistreatment in medical training.

## Additional file

Additional file 1: Case studies that were distributed to study participants. (DOCX 22 kb)

## Abbreviations

AAMC: Association of American Medical Colleges
ACGME: Accreditation Council on Graduate Medical Education
CLE: Clinical learning environment
CLER: Clinical Learning Environment Review
CORE: Culture of Respect in Education
PSCOM: Penn State College of Medicine
SLER: School of Labor and Employment Relations
