# Supplementary material for: Perceptions of mistreatment among trainees vary at different stages of clinical training
Source: BMC Med Educ. 2017 Jan 14;17:14. doi: 10.1186/s12909-016-0853-4 (PMC5237524; doi:10.1186/s12909-016-0853-4)
Supplement: Additional file 1: — Case studies that were distributed to study participants. (DOCX 22 kb) [file 12909_2016_853_MOESM1_ESM.docx]

**Additional file 1**

**CORE Case Studies**

**Scenario I: Verbal abuse, public humiliation or disrespect**

# Scenario Presentation

**Team members:** Attending Physician (Attng), senior resident (SR), medical student (MS-3), nurse.

**Time/Place:** Morning rounds, outside of the patient’s room

**Attending speaking to the MS-3:** “Can you present the patient to the team?

**MS-3:** Mrs. J has no complaints today but states she felt warm overnight. She had a fever, but is currently afebrile and her other vital signs are stable…....

**Attng:** “Fever? How do you define “a fever?” And stable? What do you mean by stable? Do you think you are experienced enough to make that judgment?” “What else?”

**MS-3**: Well, on examination her lungs were clear but the nurse thought she heard some crackles……

**Attng:** “Your presentation is out of order. I told you before that you should present in a standardized way, with every patient…… and that will teach you how to do it correctly. I don’t want to hear this kind of presentation again. ”

(Continuing)….. “Now, what do you think we should do next for this patient?” (*The attending’s facial expression shows disappointment while the remainder of the team remains silent.)*

**MS-3:** Well, I’m not sure. (*The student now appears very hesitant….)*

**Attng:** You need to put more effort into evaluating and understanding your patients. (*Turning to the* ***senior*** ***resident*** *as they walk to the bedside*)…..So what’s the real story and what are we going to do next…..

**Scenario II: Specialty-choice discrimination**

**Scenario Presentation:**

**Team members:** Pediatric hospitalist attending (Peds Attng), senior pediatric senior resident (Peds SR), PGY-1 in family and community medicine (PGY-1 FCM), PGY-1 in pediatrics (PGY-1 P), acting intern (MS-4 AI)

**Time/Place:** Daily teaching rounds in the patient’s room. It is early in the academic year and the first day that this Pediatric Hospitalist is on the service….

**Peds Attng:** Whose patient is this?

**PGY-1 FCM:** She is my patient. I have been seeing her since admission.

**Peds Attng:** Listen, this patient should really be under a pediatric intern. (To the Peds SR)… Can we make that change?

**Peds SR:** Absolutely

**PGY-1 F&CM:** But I am very familiar with her care and have a good rapport with the family.

**Peds Attng:** I am sorry, this is my decision. I think this kind of complex pediatric patient is unlikely to be cared for by someone in primary care. It would be best for this patient to be under a pediatric intern.

*During the conversation, the MS-4AI keeps silent*

*Later the same day, while checking the MS-4’s note on a patient, the* **Peds Attng** asks the MS-4 AI, “What residency are you applying for?

**MS-4 AI:** Family medicine

**Peds Attng:** Really? I think you have more potential as a subspecialist in pediatrics. In primary care you are a “jack of all trades but master of none.” Besides, the reimbursement and salaries are low and you will never pay off your debt. Think about a pediatric subspecialty before you submit that final application for residency.

**MS-4:** Ok, I’ll consider it.

*On the way out of the hospital that night, the* ***MS-4*** *complains to another medical student: “I’m so upset. I’m hesitant to tell anyone about my career choice since some attendings and residents show little respect for other areas of medicine and I am afraid it will affect my evaluations.”*

**Scenario III: Performing non-educational tasks**

**Scenario Presentation:**

**Team members:** Critical Care attending (CC Attng), anesthesia senior resident (Anesth SR), physician assistant student (PA-c student)

*During ICU rounds, the attending is called away but states he will be back in a few hours to complete rounds.*

The **Anesth SR** turns to the PA-c student and asks, “Do you have some time right now?”

**PA-c student:** Maybe a couple of minutes before I write my notes and orders, why?

**Anesth SR:** I am a little behind on my pre-op paperwork. If I give you the list can you sit down and start to complete some of the on line forms for me?

**PA-c student:** I don’t know. They aren’t my patients and I won’t know that much about them.

**Anesth SR:** Listen, there are only about 10 of them and I’ll tell you what you need to fill out. If I let the attending know that you are a “team player” I am sure it will help your evaluation for the rotation…….from both of us!

*The PA-c student completes the tasks and 2 hours later the attending returns to finish rounds in the ICU…..*

**CC Attng** to the PA-c student: Would you present the next patient to me please?

**PA-c student:** I am sorry, I was doing some chart work and I don’t have the most up to date vital signs or bloodwork results. Let me get them.

**CC Attng:** Wait, what have you been doing since I left? If you want to learn about the ICU, you should spend more time caring for the patient instead of doing those meaningless tasks that you can do after your patient care responsibilities are completed.

*The PA-c student again apologizes and the Anesth SR remains silent.*

*The* ***PA-c student*** *confides to a friend later that same day, “No one told me what my responsibilities should be and who I should listen to. I really want to get a good evaluation for this rotation and now, I don’t know what to do.”*

**Scenario IV: Withholding/denying learning opportunities**

**Scenario Presentation:**

Team: Neurosurgery fellow (Neuro Fellow), General surgery PGY-1 preliminary intern (PGY-1 prelim)

**Neuro Fellow** to PGY-1 prelim: They just called from clinic and there is a patient who is going to need a wheelchair evaluation. I think he was on our service last week and you probably know him. He needs to have it done today so go over to clinic and get that done ASAP. You can stay and see the rest of the clinic patients, also.

**PGY-1 prelim:** But my patient is about to go to the OR for a craniotomy and I want to be there.

**Neuro Fellow:** You are not a medical student. You are a resident on the team and we need to get the work done. Besides, you are going in to general surgery and it is not necessary for you to see this. Just complete the form, see the clinic patients, and we will take him to the OR. You can pick him up again when he is in the PACU.

## Scenario V: Neglect as a form of mistreatment

**Team members:** Research-clinician attending (R-C Attng), senior cardiology fellow (Cards fellow), PGY-2 internal medicine resident (PGY-2)

**Time/Location:** Department office, after evening rounds

*R-C Attng, Cards fellow, and PGY-2 resident have been engaged in a clinical research project for the entire academic year. The PGY-2 wrote the IRB proposal, has obtained and collated the data, wrote the manuscript, and has completed the forms to forward it to a prestigious peer-reviewed journal for consideration. He asked the R-C Attng and Cards fellow to review the draft and they returned it with little editing and feedback.*

**R-C Attng** to Cards Fellow and PGY-2: Well, this manuscript is very well done. The data looks good and I believe we are ready to submit. Dr. Jones (PGY-2), I believe Dr. Smith (Cards Fellow) has an excellent opportunity to get his top choice for an additional year of training in EP and if we place him as first author on this article it should seal the deal.

**PGY-2 (Dr. Jones):** But I put a lot of time into this and thought I would be first author.

**R-C Attng:** Don’t worry, you will be listed after the two of us. Remember, your time will come.

*Dr. Smith smiles and Dr. Jones is not sure what to say so he stays silent and nods his head in agreement.*

Scenario VI: Racial/gender insensitivity

**Scenario Presentation:**

**Team members:** Emergency Medicine Senior Resident (EM SR), three third year medical students (1 caucasian male, 1 Asian male – MS-3 A, and 1 female – MS-3 F), patient

**Time/Place:** In the emergency department making afternoon rounds on the active patients

In the emergency department, a senior resident who is known to be well-liked by everyone, is accompanied by 3 medical students while seeing his patients. The first patient has nausea and vomiting and he instructs the students on how to determine the appropriate rate of IVF. He asks one of the male medical students (MS-3 A) to place the IVF order in the chart. While doing so, he makes an error which the order entry system quickly “flags.” The **EM SR** looks at the student and says, “Hey, aren’t Asians supposed to be good with numbers?” He laughs and then walks toward the room of the next patient.

**EM SR** to the female medical student (MS-3 F): Please present this patient’s history while we evaluate her at the bedside.

They enter the room and the MS-3 F begins her presentation.

**MS-3 F:** This is Mrs. Brown, she is a 77 year old woman who presents after 4 hours of…..

**Patient:** (interrupting)…..Hey, I am hard of hearing. What is that *nurse* saying about me?

The **EM SR** laughs, says it is “a bunch of medical jargon” and tells the female student to continue.

When they have completed the rounds, the EM resident turns to the students and states,

**EM SR:** Why don’t you guys let me see your H&P’s for the patients you saw today and I will give you some feedback.

**MS-3 female:** Wait, what about my write ups?

**EM SR:** I don’t think I have enough time to get to everyone today but “don’t get your panties in a bunch,” I will get to you maybe tomorrow or the next day.
